# Hydrazone Derivatives Enhance Antileishmanial Activity of Thiochroman-4-ones

**DOI:** 10.3390/molecules23010070

**Published:** 2017-12-29

**Authors:** Esteban Vargas, Fernando Echeverri, Yulieth A. Upegui, Sara M. Robledo, Wiston Quiñones

**Affiliations:** 1 Química Orgánica de Productos Naturales, Instituto de Química, Facultad de Ciencias Exactas y Naturales, Universidad de Antioquia, Calle 70 No. 52–21, Medellín A. A 1226, Colombia; esteban.vargasc@udea.edu.co (E.V.); fernando.echeverri@udea.edu.co (F.E.); 2 PECET-Programa de Estudio y Control de Enfermedades Tropicales. Facultad de Medicina, Universidad de Antioquia, Calle 70 No. 52–21, Medellín A. A 1226, Colombia; yulexa1@gmail.com (Y.A.U.); sara.robledo@udea.edu.co (S.M.R.)

**Keywords:** *Leishmania*, thiochroman-4-ones, acyl hydrazone, cytotoxicity

## Abstract

Cutaneous leishmaniasis (CL) is a neglected tropical disease, which causes severe skin lesions. Due to the lack of effective vaccines, and toxicity or reduced effectiveness of available drugs in addition to complex and prolonged treatments, there is an urgent need to develop alternatives for the treatment for CL with different mechanisms of action. In our effort to search for new promising hits against *Leishmania* parasites we prepared 18 acyl hydrazone derivatives of thiochroman-4-ones. Compounds were evaluated for their in vitro antileishmanial activity against the intracellular amastigote form of *Leishmania panamensis* and cytotoxic activity against human monocytes (U-937 ATCC CRL-1593.2). Our results show that derivatization of the thiochroman-4-ones with acyl hydrazones significantly enhances the antileishmanial activity. Among the compounds tested semicarbazone and thiosemicarbazone derivatives of thioflavanone **19** and **20** displayed the highest antileishmanial activities, with EC_50_ values of 5.4 and 5.1 µM and low cytotoxicities (100.2 and 50.1 µM respectively), resulting in higher indexes of selectivity (IS).

## 1. Introduction

Leihsmaniasis is a parasitic disease caused by various species of protozoans of the genus *Leishmania* [1]. These parasites have a complex life cycle that involves an amastigote stage in the mammal host and a promastigote stage in the vector insect [1,2,3].

Cutaneous leishmaniasis (CL) causes severe skin lesions, mainly on the face, arms and feet that affect patient well-being, as well as causing severe social stigma and psychological stress. Because of the high occurrence, severe side effects of existing therapies and the lack of therapeutic alternatives the World Health Organization (WHO) considers leishmaniasis a neglected tropical disease and has encouraged countries to search for new antileishmanial drugs with novel mechanisms of action [1,4].

In our search for new chemotherapeutic alternatives to fight leishmaniasis we have explored thiochroman compounds, which could be considered a potential privileged scaffold [5,6] because of its great similarity with the chroman compounds which display a broad range of bioactivities [7]. In addition, numerous hydrazones have been reported to possess interesting biological activities such as antitumoral, antiviral and antiparasitic effects [8,9,10]; some hydrazones also exhibit activity against the parasites that cause malaria, leishmaniasis and Chagas disease [3,11,12,13,14,15,16,17,18], where the inhibition of proteases and represent the most common mechanism of action for the hydrazones [16,19,20,21].

In the search for new drugs against novel targets in *Leishmania* species Avery et al. explored the cysteine proteases inhibitors, and a total of 241,000 compounds were screened, of which 24 showed inhibition of cysteine proteases or antileishmanial activity, and 16 out of the 24 compounds possess hydrazone or imine moieties [16,17]. Semicarbazones, thiosemicarbazones and thiosemicarbazone derivatives of thiochroman-4-ones are potent inhibitors of cysteine proteases, specifically of cathepsin L [20,21,22,23,24]. However, there are no reports on the antileishmanial or cytotoxic activities of these compounds. Thus, after considering the many interesting biological activities displayed by compounds bearing the hydrazone moiety and the ability of some thiochroman-4-one thiosemicarbazone derivatives to inhibit cysteine proteases in protozoan parasites and the importance of cysteine proteases as virulence factors in *Leishmania* spp., we synthesized a series of analogues in order to screen their antileishmanial and cytotoxic activities and validate the potential of these molecules as antileishmanial candidates.

## 2. Results

### 2.1. Synthesis

**Scheme 1:**
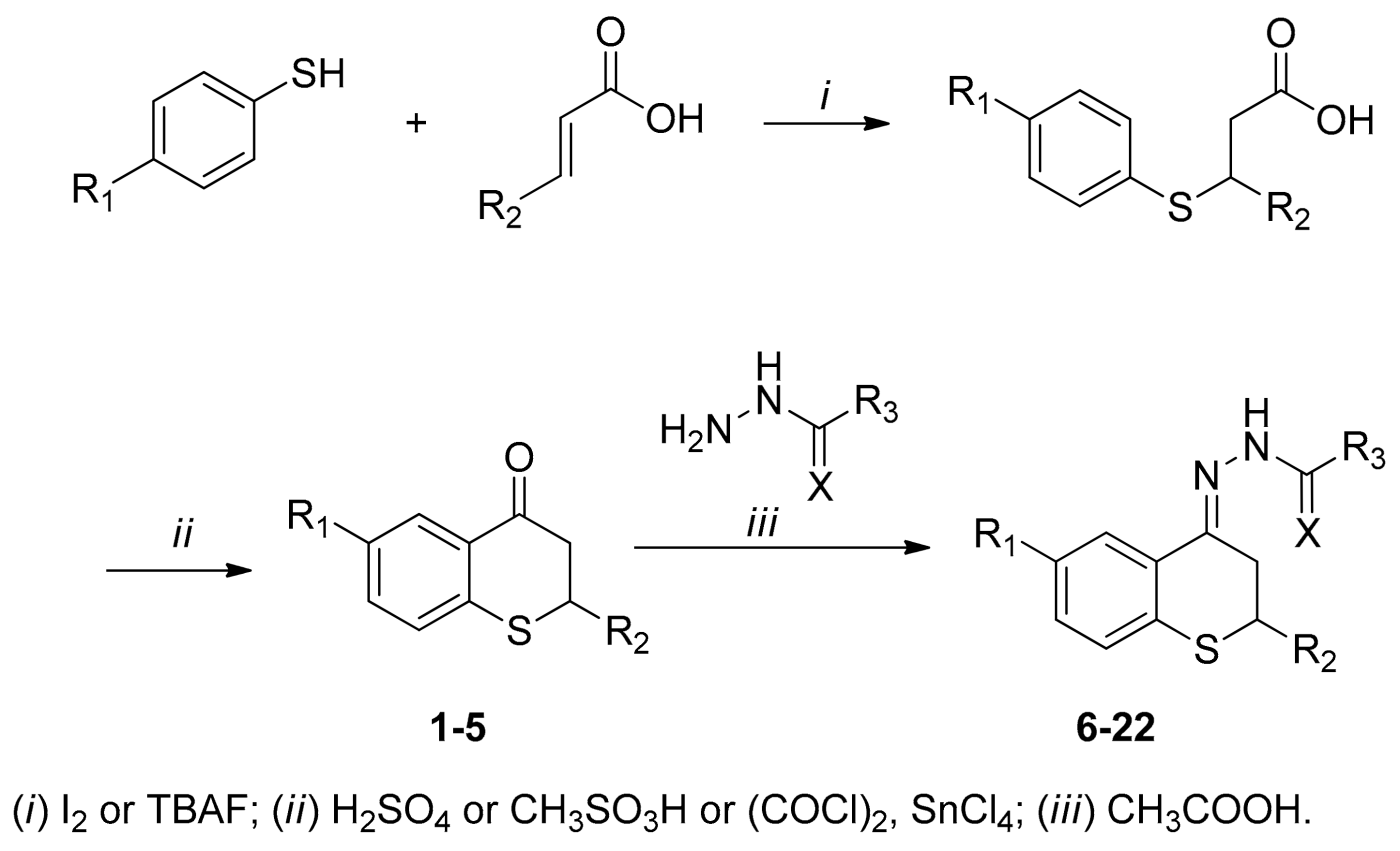
Synthesis of thiochroman-4-ones and hydrazone derivatives.

Thiochroman-4-ones were prepared by addition of thiophenol or 4-fluorothiophenol to α,β-unsaturated carboxylic acids (acrylic, crotonic or cinnamic acid) (Scheme 1). The resulting carboxylic acids undergo a ring closing reaction upon treatment with sulfuric or methanesulfonic acid to give the thiochroman-4-ones; in the case of thioflavanone ring closing reaction was carried out with oxalyl-chloride followed by tin chloride. Resultant ketones were condensed with acyl hydrazides (benzoic hydrazide, isonicotin-hydrazide, semicarbazide or thiosemicarbazide) and afforded the desired acyl-hydrazones in moderate to good yields.

### 2.2. Antileishmanial and Cytotoxic Activities

**Scheme 2:**
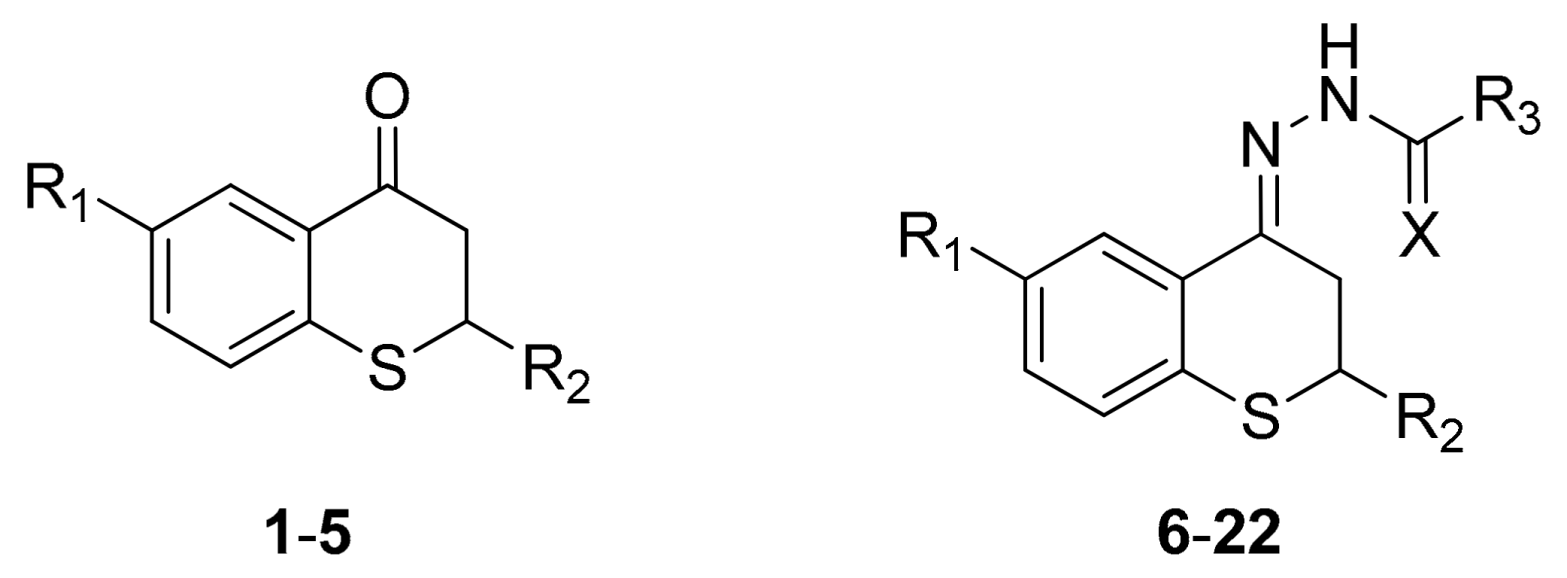
Structures of thiochroman-4-ones (**1**–**5**) and hydrazone derivatives (**6**–**22**).

All synthesized compounds were screened for their in vitro activity against intracellular amastigotes of *L. panamensis* and their cytotoxicity in human macrophages U-937. Amphotericin B was used as the control with the median effective concentration (EC_50_) and the median lethal concentration (LC_50_) values of 0.32 µM and 39.6 µM respectively (Scheme 2, Table 1). Selectivity was calculated by the ratio of LC_50_/EC_50_ and defined as index of selectivity, IS.

## 3. Discussion

EC_50_ values lower than 10 μM were selected as the minimum concentration suitable to consider a compound as promising [25], while compounds with EC_50_ values ranging from 10 to 50 μM were considered as moderately active. As can be seen in Table 1, almost all the thiochroman-4-one compounds **1**–**4** revealed weak antileishmanial activity since none of their EC_50_ values was lower than 25 µM. Thioflavanone **5** displayed moderate antileishmanial activity but also high cytotoxic activity resulting in a low IS. In general, the antileishmanial activities of hydrazones **6**–**22** were higher than those of their ketone precursors **1**–**5**; cytotoxic activity for the hydrazones has remained low, which resulted in IS higher than those of the thiochroman-4-ones. Compounds **19** and **20** showed high activity, 5.4 and 5.1 μM, respectively, while compound **22** showed also a remarkably activity with an EC_50_ of 16.4 μM and a higher index of selectivity (IS = 33.9). The presence of fluorine at C6 or methyl group at C2 did not affect the antileishmanial activity in a significant way. Replacement of the phenyl ring in R_3_ (compounds **10**, **13** and **16**) with a 4-aminophenyl ring (compounds **12**, **15** and **18**) resulted in a decrease in the antileishmanial activity. Contribution of the phenyl group in R_2_ (compounds **19**–**22**) is noteworthy, since analogous compounds **6**–**9** exhibited only marginal activities. Now, compounds **19** and **20** differ only in the replacement of the carbonyl oxygen with a larger sulfur atom, since the first is a thiosemicarbazone, while the latter is a semicarbazone, but even so, their activities are similar. On the other hand, when contrasting activity with toxicity, an excellent IS is noted, especially for **22** (>33.9) and **19** (18.6), although that of **20** is adequate (9.8); therefore, the sulfur atom seems to be more involved in the toxicity than in the leishmanicidal activity.

Although hydrazones have good stability towards hydrolysis [26] it is important to determine whether the increased of antileishmanial activity is due to the hydrazone itself or perhaps, the in situ hydrolysis products may be responsible for the increase in the activity. Garces-Eisele and Scior [27] studied the in vitro antituberculotic activity of over 200 hydrazone derivatives of isonicotinic acid hydrazide (isoniazid, INH); they found that derivatives did not improve the activity, because hydrolysis releases INH, which is the biologically active compound. In this work the antileishmanial activity of thiosemicarbazide was evaluated and compared with its hydrazones **9** and **20**; in both cases the derivative showed better activity than its precursors. In fact, the thiosemicarbazide itself was inactive against *Leishmania* parasites. Therefore, despite the available information, it cannot be deduced that acylhydrazone derivatives always act as prodrugs [28,29], since it has been shown that several hydrazones are inhibitors of cysteine proteases [16,20,21].

Moreover, Song et al. [21] showed that semicarbazones of some thiochroman-4-ones inhibit cathepsin L, which is structurally related to the cysteine protease cruzain, a common protease in trypanosomatid parasites. Further experiments are necessary to determine if the mechanism of actions of these hydrazones may, in whole or part, be due to the inhibition of cysteine proteases in *Leishmania*.

## 4. Materials and Methods

### 4.1. Chemistry

#### 4.1.1. General

All commercially available reagents and solvents were obtained from commercial suppliers and used without further purification. Commercial thiocroman-4-one **1** (97%) was purchased from Sigma Chemical Co. (St. Louis, MO, USA). The reaction progress was monitored with thin layer chromatography on silica gel TLC aluminum sheets (60F_254_, Merck, Darmstadt, Germany). The melting points were determined using a Mel-Temp apparatus (Electrothermal, Staffordshire, UK) and are uncorrected. FTIR spectra were obtained on a Bruker Alpha FTIR spectrometer (Bruker Optic GmbH, Ettlingen, Germany). ^1^H- and ^13^C-nuclear magnetic resonance (NMR) spectra were recorded using Bruker Fourier 300 spectrometer (Bruker Bio-Spin GmbH, Rheinstetten, Germany) operating at 300 MHz for ^1^H and 75 MHz for ^13^C. Samples were dissolved in DMSO-*d*_6_ or CDCl_3_ using TMS as internal standard. HRMS was obtained using Q-ToF quadrupole/orthogonal spectrometry (Waters, Milford, MA, USA) in either negative (reported as [M − H]^−^) or positive mode (reported as [M + H]^+^) and Bruker Impact II UHR-Q-TOF mass spectrometer (Bruker Daltonik, Bremen, Germany) in positive mode.

#### 4.1.2. Synthesis of Thiochroman-4-ones and Thioflavanone

*6-Fluorothiochroman-4-one* (**2**). To a mixture of acrylic acid (700 µL, 720 mg, 10 mmol) and 4-fluorothiophenol (1985 mg, 15 mmol) was added I_2_ (20% mol, 760 mg, 3 mmol) and the mixture was stirred at 50 °C for 24 h. After completion of reaction (monitored by TLC), a cold saturated sodium thiosulfate solution (30 mL) was added and extracted with dichloromethane (2 × 25 mL); the combined organic layers were mixed with a saturated solution of sodium bicarbonate and extracted to remove the unreacted starting material. The aqueous layer was acidified with 10% HCl and extracted with dichloromethane (3 × 50 mL). The combined organic layers were dried over Na_2_SO_4_, evaporation of the solvent under reduced pressure afforded 1150 mg (64%) of the desired addition product. The product was cooled down to 0 °C in an ice bath and 3 mL of concentrated sulfuric acid was added and the reaction mixture was allowed to warm to room temperature for 2 h with magnetic stirring. The reaction was quenched with ice and the mixture was extracted with dichloromethane (3 × 50 mL). The combined organic layers were washed once with water, followed by saturated NaHCO_3_ solution. The combined organic layers were washed with brine, dried over Na_2_SO_4_, and concentrated under reduced pressure. The residue was purified by column chromatography over silica gel using hexane/EtOAc (9:1) as eluent to give 570 mg (90%) of pure **2** as a yellow solid. m.p.: 86–88 °C. ^1^H-NMR (CDCl_3_) δ 8.07 (dd, *J* = 8.0, 1.4 Hz, 1H), 7.16–7.11 (m, 2H), 3.25–3.23 (m, 2H), 2.99–2.96 (m, 2H). ^13^C-NMR (CDCl_3_) δ 193.5, 160.9 (d, *J_C–F_* = 245 Hz), 137.8, 132.7, 129.8 (d, *J_C–F_* = 7 Hz), 121.70 (d, *J_C–F_* = 23.0 Hz), 115.6 (d, *J_C–F_* = 22.6 Hz), 39.7, 27.1. IR ν: 1659, 1595, 1565. HRMS (ESI) calculated for C_9_H_6_FOS [M − H]^−^ 181.0123, found 181.0165.

*2-Methylthiochroman-4-one* (**3**). To a mixture of crotonic acid (860 mg, 10 mmol) and thiophenol (1.650 g, 15 mmol) was added I_2_ (20% mol, 255 mg, 1 mmol) and the mixture was stirred at room temperature for 12 h. After completion of the reaction (monitored by TLC), a cold saturated sodium thiosulfate solution (20 mL) was added and extracted with dichloromethane (2 × 50 mL); then, combined organic layers were mixed with a saturated solution of sodium bicarbonate and extracted to remove the unreacted starting material. The aqueous layer was acidified with 10% HCl and extracted with dichloromethane (3 × 40 mL). The combined organic layers were dried over Na_2_SO_4_, evaporation of the solvent under reduced pressure afforded 1.962 g (86%) of the desired addition product. After, 200 mg (1.0 mmol) of this product were cooled down to 0 °C in an ice bath and 3.0 mL of concentrated sulfuric acid was added; the reaction mixture was stirred for 30 min, and, after that, the ice bath was removed allowing the reaction mixture to warm to room temperature for another 2 h under continuous stirring. The reaction was quenched with ice and the mixture was extracted with dichloromethane (3 × 25 mL). The combined organic layers were washed once with water, followed by addition of a saturated NaHCO_3_ solution. The combined organic layers were dried over Na_2_SO_4_, and concentrated under reduced pressure. The residue was purified by column chromatography over silica gel using hexane:EtOAc (9:1) as eluent, to give 137 mg (75%) of pure **3** as a yellowish oil. ^1^H-NMR (CDCl_3_) δ 8.08 (d, *J* = 8.0 Hz, 1H), 7.38 (t, *J* = 7.5 Hz, 1H), 7.25 (d, *J* = 7.8 Hz, 1H), 7.16 (t, *J* = 7.6 Hz, 1H), 3.74–3.53 (m, 1H), 2.98 (dd, *J* = 17.6, 8.8 Hz, 1H), 2.84–2.66 (m, 1H), 1.43 (d, *J* = 6.8 Hz, 3H). ^13^C-NMR (CDCl_3_) δ 194.9, 141.9, 133.7, 130.1, 129.1, 127.65, 125.1, 48.0, 36.5, 20.6. IR ν: 2964, 1679, 1587. HRMS (ESI) calculated for C_10_H_11_OS [M + H]^+^ 179.0525, found 179.0536.

*6-Fluoro-2-methylthiochroman-4-one* (**4**). To a mixture of crotonic acid (172 mg, 2 mmol) and 4-fluorothiophenol (385 mg, 3.0 mmol) was added I_2_ (20% mol, 52 mg, 0.2 mmol) and the mixture was stirred at room temperature for 12 h. After completion of reaction (monitored by TLC), a cold saturated sodium thiosulfate solution (20 mL) was added and extracted with dichloromethane (2 × 25 mL); the combined organic layers were mixed with a saturated solution of sodium bicarbonate and extracted to remove the unreacted starting material. The aqueous layer was acidified with 10% HCl and extracted with dichloromethane (3 × 25 mL). The combined organic layers were dried over Na_2_SO_4_, evaporation of the solvent under reduced pressure afforded 200 mg (94%) of the addition product. Thus, compounds were cooled down to 0 °C in an ice bath and 2.0 mL of concentrated sulfuric acid was added and the reaction mixture was allowed to warm to room temperature for 2 h with continuous stirring. The reaction was quenched with ice and the mixture was extracted with dichloromethane (3 × 25 mL). The combined organic layers were washed once with water, followed by saturated NaHCO_3_ solution. The combined organic layers were washed with brine, dried over Na_2_SO_4_, and concentrated under reduced pressure. The residue was purified by column chromatography over silica gel using hexane/EtOAc (9:1) as eluent to give 128 mg (64%) of pure **4** as a yellowish oil. ^1^H-NMR (CDCl_3_) δ 7.84–7.72 (m, 1H), 7.39–6.97 (m, 2H), 3.78–3.47 (m, 1H), 3.07–2.95 (m, 1H), 2.80–2.68 (m, 1H), 1.43 (d, *J* = 6.9 Hz, 3H). ^13^C-NMR (CDCl_3_) δ 194.2, 160.7 (d, *J_C-F_* = 246 Hz), 137.6, 132.3, 129.7 (d, *J_C-F_* = 7.0 Hz), 121.8 (d, *J_C-F_* = 23.1 Hz), 115.4 (d, *J_C-F_* = 22.8 Hz), 47.9, 37.0, 20.7. IR ν: 2967, 1684, 1602. HRMS (ESI) calculated for C_10_H_10_FOS [M + H]^+^ 197.0431, found 197.0443.

*2-Phenylthiochroman-4-one* (thioflavanone) (**5**). Cinnamic acid (297 mg, 2 mmol) and thiophenol (330 mg, 3 mmol) were mixed with 75% aqueous solution of TBAF (140 µL sln, 0.4 mmol) and the mixture was stirred for 4 h at 60 °C. A saturated solution of sodium bicarbonate was added and extracted with dichloromethane (3 × 25 mL) to remove the unreacted starting material. The aqueous layer was acidified with 10% HCl and extracted with dichloromethane (3 × 30 mL). The combined organic layers were dried over Na_2_SO_4_; evaporation of the solvent under reduced pressure gave the crude addition product which was dissolved in anhydrous dichloromethane and placed in an oven-dried round bottomed flask under N_2_ in an ice cooling bath. Consequently, oxalyl chloride (365 µL, 3.0 mmol) was added dropwise followed by two drops of DMF and the reaction mixture is left to warm to room temperature. After stirring for 2.5 h, the solution was cooled to −10 °C, and a solution of 1 M SnCl_4_ (3.0 mL, 3.0 mmol) in CH_2_Cl_2_ was added dropwise. The resulting mixture was stirred at 0 °C for 10 min and then allowed to warm to room temperature. After stirring at room temperature for 12 h, water (25 mL) was added and extracted with dichloromethane (3 × 25 mL). The combined organic layers dried over anhydrous Na_2_SO_4_, and concentrated under reduced pressure. The residue was purified by column chromatography over silica gel using hexane/EtOAc (2:1) as eluent to give the desired thioflavanone **5**. Yield 215 mg (45%) white solid. m.p. 155–157 °C. ^1^H-NMR (CDCl_3_) δ 8.20 (dd, *J* = 7.9, 1.2 Hz, 1H), 7.52–7.36 (m, 6H), 7.35–7.30 (m, 1H), 7.30–7.21 (m, 1H), 4.77 (dd, *J* = 12.7, 3.3 Hz, 1H), 3.56–3.07 (m, 2H). IR ν: 1665, 1586, 1556, 1452, 1433. HRMS (ESI) calculated for C_15_H_13_OS [M + H]^+^ 241.0687, found 241.0694.

#### 4.1.3. General Procedure for the Preparation of Acyl Hydrazone Derivatives

Thiochroman-4-ones (0.5 mmol) were dissolved in anhydrous methanol (25 mL). The mixture was heated at reflux and then hydrazide (1.0 mmol, 2 equiv.) and glacial acetic acid 60 µL were added. After 12 h at reflux, the resulting precipitate was collected through filtration and washed with methanol. After drying under vacuum, the residue was passed through a small pad of silica gel with ethyl acetate, after evaporation of the solvent acyl hydrazones were obtained as white solids.

*(E)-N′-(Thiochroman-4-ylidene)benzohydrazide* (**6**). Yield 80%, m.p.: 160–161 °C. ^1^H-NMR (DMSO-*d*_6_) δ 10.79 (s, 1H), 8.20 (br s, 1H), 7.87 (br s, 2H), 7.54 (d, *J* = 7.8 Hz, 1H), 7.52 (d, *J* = 7.8 Hz, 2H), 7.24 (m, 2H), 7.16 (br s, 1H), 3.07 (br s 4H). ^13^C-NMR (CDCl_3_) δ 164.5, 153.1, 136.8, 134.5, 132.0, 129.9, 128.8, 128.40, 128.39, 121.8, 126.0, 125.7, 25.7, 28.4. HRMS (ESI) calculated for C_16_H_15_N_2_OS [M + H]^+^ 283.0900 found 283.0906. IR ν: 2997, 1645, 1597, 1532, 1275, 1136.

*(E)-N′-(Thiochroman-4-ylidene)isonicotinohydrazide* (**7**). Yield 60%, m.p.: 175–176 °C. ^1^H-NMR (DMSO-*d*_6_) δ 11.07 (s, 1H), 8.77 (d, *J* = 5.6 Hz, 2H), 8.17 (d, *J* = 7.5 Hz, 1H), 7.80 (d, *J* = 3.7 Hz, 2H), 7.30 (q, *J* = 7.6 Hz, 2H), 7.25–7.15 (m, 1H), 3.10 (s, 4H). ^13^C-NMR (DMSO-*d*_6_) δ 162.5, 154.1, 150.2, 141.0, 136.6, 131.0, 129.9, 128.0, 127.0, 125.3, 121.9, 28.1, 25.1. HRMS (ESI) calculated for C_15_H_14_N_3_OS [M + H]^+^ 284.0852 found 284.0864. IR ν: 2924, 1637, 1597, 1530, 761.

*(E)-2-(Thiochroman-4-ylidene)hydrazinecarboxamide* (**8**) Yield 79%, m.p.: 214–216 °C. ^1^H-NMR (DMSO-*d*_6_) δ 9.34 (s, 1H), 8.21 (d, *J* = 7.8 Hz, 1H), 7.24–7.03 (m, 3H), 6.56 (s, 2H), 3.15–2.90 (m, 2H), 2.91–2.72 (m, 2H). ^13^C-NMR (DMSO-*d*_6_) δ 157.7, 142.4, 135.5, 132.2, 128.9, 128.3, 127.1, 125.8, 28.2, 25.7. HRMS (ESI) calculated for C_10_H_12_N_3_OS [M + H]^+^ 222.0696 found 222.0703. IR ν: 3456, 3186, 1697, 1584, 1430, 1248, 1201.

*(E)-2-(Thiochroman-4-ylidene)hydrazinecarbothioamide* (**9**). Yield 55%, m.p.: 228–230 °C. ^1^H-NMR (DMSO-*d*_6_) δ 10.21 (s, 1H), 8.32 (d, *J* = 8.0 Hz, 2H), 8.00 (s, 1H), 7.27–7.19 (m, 2H), 7.12 (ddd, *J* = 8.3, 6.1, 2.5 Hz, 1H), 3.01 (s, 4H).^13^C-NMR (DMSO-*d*_6_) δ 179.3, 145.8, 136.6, 131.4, 129.7, 128.3, 127.8, 125.7, 28.4, 25.5. HRMS (ESI) calculated for C_10_H_12_N_3_S_2_ [M + H]^+^ 238.0467 found 238.0482. IR ν: 3410, 3126, 1596, 1468, 1285.

*(E)-N′-(6-Fluorothiochroman-4-ylidene)benzohydrazide* (**10**). Yield 76%, m.p.: 168 °C. ^1^H-NMR (DMSO-*d*_6_) δ 10.91 (s, 1H), 8.02–7.77 (m, 3H), 7.58 (dd, *J* = 15.3, 8.6 Hz, 1H), 7.52 (t, *J* = 7.3 Hz, 2H), 7.33 (dd, *J* = 8.9, 5.2 Hz, 1H), 7.18 (t, *J* = 8.0 Hz, 1H), 3.10 (br s, *J* = 24.2 Hz, 4H). ^13^C-NMR (DMSO-*d*_6_) δ 164.2, 159.9 (d, *J* = 241.4 Hz), 151.0, 133.9, 133.2 (d, *J* = 7.3 Hz), 131.9, 131.7, 130.0 (d, *J* = 7.7 Hz), 128.4 (s), 128.1, 117.1 (d, *J* = 18.7 Hz), 112.5 (d, *J* = 24.1 Hz), 27.6, 25.21. HRMS (ESI) calculated for C_16_H_14_FN_2_OS [M + H]^+^ 301.0805 found 301.0820. IR ν: 1666, 1643, 1537, 1282, 1138.

*(E)-N′-(6-Fluorothiochroman-4-ylidene)isonicotinohydrazide* (**11**). Yield 53%, m.p.: 182–183 °C. ^1^H-NMR (DMSO-*d*_6_) δ 11.14 (s, 1H), 8.77 (br s, 2H), 7.89 (d, *J* = 10.5 Hz, 1H), 7.81 (br s, 2H), 7.33 (dd, *J* = 12.1, 6.3 Hz, 1H), 7.21 (t, *J* = 9.5 Hz, 1H), 3.10 (br s, 4H). ^13^C-NMR (DMSO-*d*_6_) δ 162.8, 159.9 (d, *J* = 242.6 Hz), 152.4, 150.2, 140.9, 132.9, 132.3, 130.1 (d, *J* = 7.6 Hz), 122.0, 117.5 (d, *J* = 22.8 Hz), 112.7 (d, *J* = 23.7 Hz), 27.8, 25.2. HRMS (ESI) calculated for C_15_H_13_FN_3_OS [M + H]^+^ 302.0758 found 302.0773. IR ν: 3461, 3100, 2920, 1666, 1539, 1281.

*(E)-4-Amino-N′-(6-fluorothiochroman-4-ylidene)benzohydrazide* (**12**). Yield 82%, m.p.: 223–224 °C. ^1^H-NMR (DMSO-*d*_6_) δ 10.40 (s, 1H), 7.84 (d, *J* = 10.8 Hz, 1H), 7.64 (d, *J* = 8.5 Hz, 2H), 7.30 (dd, *J* = 8.7, 5.6 Hz, 1H), 7.15 (td, *J* = 8.4, 2.9 Hz, 1H), 6.60 (d, *J* = 8.6 Hz, 2H), 5.75 (d, *J* = 17.4 Hz, 2H), 3.07 (s, 4H). ^13^C-NMR (DMSO-*d*_6_) δ 164.7, 160.1 (d, *J* = 241.1 Hz), 152.5, 148.5, 133.6 (d, *J* = 7.2 Hz), 131.7 (d, *J* = 2.5 Hz), 130.1 (d, *J* = 7.5 Hz), 130.0, 119.7, 116.8 (d, *J* = 22.5 Hz), 112.7, 112.5 (d, *J* = 22.3 Hz), 27.5, 25.4. HRMS (ESI) calculated for C_16_H_15_FN_3_OS [M + H]^+^ 316.0914 found 316.0933. IR ν: 3480, 3332, 1647, 1634, 1614, 1594, 1533, 1507, 1267, 1148.

*(E)-N′-(2-Methylthiochroman-4-ylidene)benzohydrazide* (**13**). Yield 79%, m.p.: 205–206 °C. ^1^H-NMR (DMSO-*d*_6_) δ 10.86 (s, *J* = 46.9 Hz, 1H), 8.18 (br s, 1H), 7.88 (br s, 2H), 7.64–7.45 (m, *J* = 14.3, 6.8 Hz, 3H), 7.34–7.14 (m, 3H), 3.58–3.32 (m, 2H), 2.70 (dd, *J* = 17.1, 10.8 Hz, 1H), 1.36 (d, *J* = 6.7 Hz, 3H). ^13^C-NMR (DMSO-*d*_6_) δ 164.0, 152.7, 135.8, 134.0, 131.6, 130.8, 129.8, 128.3, 128.0, 127.8, 126.7, 125.2, 35.6, 34.8, 20.3. HRMS (ESI) calculated for C_17_H_17_N_2_OS [M + H]^+^ 297.1056 found 297.1067. IR ν: 3217,1665, 1527, 1276, 1132.

*(E)-N′-(2-Methylthiochroman-4-ylidene)isonicotinohydrazide* (**14**). Yield 65%, m.p.: 194–195 °C. ^1^H-NMR (DMSO-*d*_6_) δ 9.57 (s, 1H), 8.90–8.75 (m,2H), 7.85–7.71 (m, 2H), 7.05–7.40 (m, 4H), 3.57–3.36 (m, 1H), 3.21 (dd, *J* = 16.4, 3.0 Hz, 1H), 2.66 (dd, *J* = 16.4, 11.0 Hz, 1H), 1.50 (d, *J* = 6.7 Hz, 3H). ^13^C-NMR (DMSO-*d*_6_) δ 162.6, 154.1, 150.2, 141.0, 136.1, 130.5, 130.1, 127.8, 126.8, 125.2, 122.0, 35.7, 34.7, 20.3. HRMS (ESI) calculated for C_16_H_16_N_3_OS [M + H]^+^ 298.1009 found 298.1024. IR ν: 3174, 2962, 1650, 1600, 1529, 1281.

*(E)-4-Amino-N′-(2-methylthiochroman-4-ylidene)benzohydrazide* (**15**). Yield 60%, m.p.: 168–169 °C. ^1^H-NMR (DMSO-*d*_6_) δ 10.35 (s, 1H), 8.12 (d, *J* = 7.8 Hz, 1H), 7.65 (d, *J* = 8.5 Hz, 2H), 7.38–6.97 (m, 3H), 6.60 (d, *J* = 8.6 Hz, 2H), 5.74 (d, *J* = 9.2 Hz, 2H), 3.65–3.11 (m, 2H), 2.67 (dd, *J* = 16.8, 10.4 Hz, 1H), 1.35 (d, *J* = 6.6 Hz, 3H). ^13^C-NMR (DMSO-*d*_6_) δ 164.3, 152.4, 150.5, 135.5, 131.2, 130.1, 129.6, 127.9, 126.6, 125.3, 119.9, 112.7, 35.4, 34.9, 20.5. HRMS (ESI) calculated for C_17_H_18_N_3_OS [M + H]^+^ 312.1165 found 312.1187. IR ν: 3473, 3360, 2921, 1634, 1613, 1612, 1372.

*(E)-N′-(6-Fluoro-2-methylthiochroman-4-ylidene)benzohydrazide* (**16**). Yield 52%, m.p.: 206–207 °C. ^1^H-NMR (DMSO-*d*_6_) δ 10.92 (s, 1H), 8.03–7.75 (m, 3H), 7.65–7.46 (m, 3H), 7.30 (dd, *J* = 8.8, 5.4 Hz, 1H), 7.25–7.15 (m, 1H), 3.50–3.27 (m, 2H), 2.69 (dd, *J* = 17.1, 10.9 Hz, 1H), 1.35 (d, *J* = 6.6 Hz, 3H). ^13^C-NMR (DMSO-*d*_6_) δ 164.2, 159.9 (d, *J* = 241.4 Hz), 151.1, 133.9, 132.7 (d, *J* = 7.3 Hz), 131.7, 131.3, 129.8 (d, *J* = 7.7 Hz), 128.3, 128.1, 117.3 (d, *J* = 23.0 Hz), 112.3 (d, *J* = 24.3 Hz), 35.3, 34.8, 20.2. HRMS (ESI) calculated for C_17_H_16_FN_2_OS [M + H]^+^ 315.0962 found 315.0977. IR ν: 3235, 1667, 1526, 1280, 1132.

*(E)-N′-(6-Fluoro-2-methylthiochroman-4-ylidene)isonicotinohydrazide* (**17**). Yield 85%, m.p.: 210–212 °C. ^1^H-NMR (DMSO-*d*_6_) δ 11.16 (s, 1H), 8.77 (br s, 2H), 7.89 (d, *J* = 10.1 Hz, 1H), 7.80 (br s, 2H), 7.29 (dd, *J* = 17.4, 11.5 Hz, 1H), 7.21 (t, *J* = 9.1 Hz, 1H), 3.51 – 3.22 (m, *J* = 28.2 Hz, 2H), 2.69 (dd, *J* = 17.0, 11.0 Hz, 1H), 1.35 (d, *J* = 6.5 Hz, 3H). ^13^C-NMR (DMSO-*d*_6_) δ 162.9, 159.9 (d, *J* = 241.2 Hz), 152.5, 150.2, 140.9, 132.4 (d, *J* = 7.3 Hz), 131.7, 129.9 (d, *J* = 7.6 Hz), 122.1, 117.7 (d, *J* = 22.8 Hz), 112.5 (d, *J* = 23.3 Hz), 35.5, 34.9, 20.2. HRMS (ESI) calculated for C_16_H_15_FN_3_OS [M + H]^+^ 316.0914 found 316.0923. IR ν: 3183, 1646, 1376, 1269.

*(E)-4-Amino-N′-(6-fluoro-2-methylthiochroman-4-ylidene)benzohydrazide* (**18**). Yield 60%, m.p.: 187–189 °C. ^1^H-NMR (DMSO-*d*_6_) δ 10.42 (s, 1H), 7.85 (dd, *J* = 11.0, 3.2 Hz, 1H), 7.65 (s, 2H), 7.27 (dt, *J* = 10.1, 5.0 Hz, 1H), 7.16 (tt, *J* = 8.6, 4.3 Hz, 1H), 6.60 (d, *J* = 8.6 Hz, 2H), 5.79 (s, *J* = 19.0 Hz, 1H), 3.58–3.14 (m, 2H), 2.67 (dd, *J* = 17.1, 10.4 Hz, 1H), 1.35 (d, *J* = 6.7 Hz, 3H). ^13^C-NMR (DMSO-*d*_6_) δ 164.9, 160.1 (d, *J* = 241.3 Hz), 152.5, 148.7, 133.2 (d, *J* = 7.5 Hz), 131.0 (d, *J* = 2.4 Hz), 130.2, 129.9 (d, *J* = 7.5 Hz), 119.7 (s), 117.1 (d, *J* = 22.4 Hz), 112.7, 112.3 (d, *J* = 23.8 Hz), 35.1, 35.0, 20.3. HRMS (ESI) calculated for C_17_H_17_FN_3_OS [M + H]^+^ 330.1071 found 330.1093. IR ν: 3381, 3065, 2955, 1623, 1604, 1566, 1372.

*(E)-2-(2-Phenylthiochroman-4-ylidene)hydrazinecarboxamide* (**19**). Yield 47%, m.p.: 194–195 °C. ^1^H-NMR (DMSO-*d*_6_) δ 9.46 (s, 1H, NH), 8.26 (d, *J* = 7.7 Hz, 1H, H5), 7.49 (d, *J* = 7.3 Hz, 2H), 7.35 (dt, *J* = 19.1, 7.0 Hz, 3H), 7.28–7.09 (m, 3H), 6.56 (s, 2H), 4.51 (dd, *J* = 11.9, 3.0 Hz, 1H, H2), 3.56, (m, 1H, H3), 3.03 (dd, *J* = 17.4, 12.0 Hz, 1H, H3). ^13^C-NMR (DMSO-*d*_6_) δ 157.6, 142.7, 139.9, 135.3, 131.9, 129.2, 129.1, 128.4, 128.1, 128.0, 127.0, 126.1, 43.9, 34.8. HRMS (ESI) calculated for C_16_H_16_N_3_OS [M + H]^+^ 298.1009 found 298.1024. IR ν: 3465, 3136, 1696, 1664, 1412, 746.

*(E)-2-(2-Phenylthiochroman-4-ylidene)hydrazinecarbothioamide* (**20**). Yield 38%, m.p.: 198–200 °C. ^1^H-NMR (DMSO-*d*_6_) δ 10.42 (s, 1H,), 8.39 (d, *J* = 7.8 Hz, 1H), 8.29 (s, 1H), 8.04 (s, 1H), 7.74–7.21 (m, 7H), 7.17 (t, 1H) 4.52 (d, *J* = 9.8 Hz, 1H), 3.54–3.51 (m, 1H), 3.16 (dd, *J* = 17.4, 12.4 Hz, 1H). ^13^C-NMR (DMSO-*d*_6_) δ 179.4, 146.0, 139.7, 136.4, 131.1, 130.0, 129.1, 128.5, 128.1, 128.0, 127.7, 126.0, 43.8, 35.1. HRMS (ESI) calculated for C_16_H_16_N_3_S_2_ [M + H]^+^ 314.0780 found 314.0779. IR ν: 3423, 3240, 2925, 1593, 1504, 1461.

*(E)-N′-(2-Phenylthiochroman-4-ylidene)benzohydrazide* (**21**). Yield 45%, m.p.: 232–233 °C. ^1^H-NMR (DMSO-*d*_6_) δ 10.90 (s, 1H, NH), 7.81 (d, *J* = 7.2 Hz, 2H), 7.64–7.15 (br m, 12H), 4.57 (dd, *J* = 12.3, 2.9 Hz, 1H), 3.57 (m, 1H), 3.21 (dd, *J* = 17.2, 12.4 Hz, 1H). ^13^C-NMR (DMSO-*d*_6_) δ 164.9 (C=O), 152.7 (C=N), 139.9, 136.6, 134.2, 132.1, 131.3, 130.3, 129.2, 128.8, 128.6, 128.4, 128.1, 128.0, 127.3, 126.0, 44.1 (C3), 35.2 (C2). HRMS (ESI) calculated for C_22_H_19_N_2_OS [M + H]^+^ 359.1213 found 359.1232. IR ν: 3059, 1633, 1567, 763.

*(E)-N′-(2-Phenylthiochroman-4-ylidene)isonicotinohydrazide* (**22**). Yield 45%, m.p.: 240–242 °C. ^1^H-NMR (DMSO-*d*_6_) δ 11.12 (s, 1H), 8.71 (d, *J* = 4.0 Hz, 2H), 8.23 (d, *J* = 7.7 Hz, 1H), 7.73 (d, *J* = 3.8 Hz, 2H), 7.50–7.10 (m, 8H), 4.58 (dd, *J* = 12.3, 2.7 Hz, 1H), 3.58 (d, *J* = 16.1 Hz, 1H), 3.20 (dd, *J* = 16.8, 12.8 Hz, 1H). ^13^C-NMR (DMSO-*d*_6_) δ 163.5, 153.9, 150.5, 141.5, 139.8, 136.9, 131.1, 130.6, 129.2, 128.6, 128.2, 128.1, 127.4, 126.0, 122.4, 44.0, 35.4. HRMS (ESI) calculated for C_21_H_18_N_3_OS [M + H]^+^ 360.1165 found 360.1184. IR ν: 3167, 1635, 1534, 756.

### 4.2. Biological Activity

#### 4.2.1. Cytotoxic Activity

Cytotoxicity of the compounds was evaluated over human monocytes (U-937 ATCC CRL-1593.2) in exponential growing phase and, adjusted at 1 × 10^5^ cells/mL in RPMI-1640 (GIBCO Invitrogen, Basel, Switzerland) enriched with 10% fetal bovine serum (FBS). One hundred microliters of cell suspension were dispensed in each well of a 96-wells microplate and then, 100 μL of each compound or standard drug (amphotericin B) at four serial dilution concentrations (200, 50, 12.5 and 3.125 μg/mL) were added dissolved in phosphate buffer solution (PBS) with 0.5% dimethyl sulfoxide. Cells exposed to compounds or standard drug were incubated 72 h at 37 °C and 5% of CO_2_. Cytotoxic activity of each compound was determined according to the effect on the cell viability by the MTT microenzymatic method in which 3-(4,5-dimethylthiazol-2-yl)-2,5-diphenyltetrazolium bromide is reduced to a purple product named formazan by mitochondrial enzyme succinate dehydrogenase. Thus, 10 μL/well of MTT solution (5 mg/mL) were added to each well of exposed and unexposed cells, and plates were incubated at 37 °C, 5% CO_2_ during 3 h. The reaction was stopped by adding 100 μL/well of isopropanol with 50% and 10% of sodium dodecyl sulfate. The concentration of formazan was determined spectrophotometrically at 570 nm (Varioskan Flash Multimode Reader, Thermo Scientific, Waltham, MA, USA) and intensity of color (absorbance) was registered as O.D. Cells exposed to amphotericin B were used as control for toxicity (positive control) while cell incubated in absence of any compound or drug were used as control for viability (negative control). Non-specific absorbance was corrected by subtracting absorbance (O.D.) of the blank. Determinations were done by triplicate in at least two independent experiments [30].

#### 4.2.2. Antileishmanial Activity

Antileishmanial activity of compounds was determined according to the ability to reduce the infection by *L. panamensis* parasites, a *Leishmania* species that is highly prevalent in the Central America and Colombia as causative agent of C.L. For this, the antileishmanial activity was tested on intracellular amastigotes of *L. panamensis* transfected with the green fluorescent protein gene (MHOM/CO/87/UA140-EGFP strain) [31]. Briefly, U-937 human cells at a density of 3 × 10^5^ cells/mL in RPMI 1640 and 0.1 μg/mL of phorbol-12-myristate-13-acetate were dispensed on 24-wells microplate and then infected with stationary phase growing *L. panamensis* promastigotes in 15:1 parasites per cell ratio. Plates were incubated at 34 °C and 5% CO_2_ for 3 h and then cells were washed twice with PBS to eliminate not internalized parasites. Fresh RPMI-1640 was added into each well (1 mL) and plates were incubated again. After 24 h of infection, the RPMI-1640 medium was replaced by fresh culture medium containing each compound at four serial dilutions (50, 12.5, 3.125 and 0.78 μg/mL) and plates were then incubated at 37 °C and 5% CO_2_ during 72 h, then, cells were removed from the bottom plate with 100 μL of EDTA/trypsin (250 mg) solution. The cells were centrifuged at 168× *g* during 10 min at 4 °C, the supernatant was discarded and cells were washed with 1 mL of cold PBS and centrifuged at 168× *g* for 10 min at 4 °C. Cells were washed two times employing PBS, as previously, and after the last wash, the supernatant was discarded and cells were suspended in 500 μL of PBS.

Cells were analyzed by flow cytometry employing a flow cytometer (Cytomics FC 500MPL, Beckman Coulter. Pasadena, CA, USA) reading at 488 nm (exciting) and 525 nm (emitting) over an argon laser and counting 10,000 events. Infected cells were determined according the events for green fluorescence (parasites). All determinations for each compound and standard drug were carried out by triplicate, in two experiments. Infected cells exposed to amphotericin B were used as control for antileishmanial activity (positive control) while infected cells incubated in absence of any compound or drug were used as control for infection (negative control). Nonspecific fluorescence was corrected by subtracting fluorescence of unstained cells. Determinations were done by triplicate in at least two independent experiments [31,32].

#### 4.2.3. Statistical Analysis

Cytotoxicity was determined according to viability and mortality percentages obtained for each experimental condition (synthetized compounds, amphotericin B and culture medium). Results were expressed as the LC_50_, concentration necessary to kill 50% of cells, calculated by the parametric method of linear regression that permits the construction of a doses-response and the calculation of the LC_50_ (Probit analysis) [32]. Initially, viability percentages were calculated by Equation (1), where the O.D. of control well, corresponds to 100% of viability:

% viability = (O.D. exposed cells/O.D. unexposed cells) × 100(1)

Then, the percentage of cell growth inhibition was calculated using Equation (2):

% inhibition = 100 − (% viability)(2)

The toxicity was defined according to LC_50_ values, using the following scale: High Toxicity; LC_50_ < 100 μM; moderate toxicity; LC_50_ > 100 μM and <200 μM and potential nontoxicity; LC_50_ > 200 μM.

Antileishmanial activity was determined according reduction of percentage of fluorescent parasites determined according to the median fluorescence intensity (MFI), obtained for each experimental condition by cytometry. The parasite values for each concentration of compound were calculated by Equation (3), where the % of parasites in the control well, corresponds to 100% of parasites:

% parasites = (MFI exposed parasites/MFI unexposed parasites) × 100(3)

Then, inhibition percentage was calculated with Equation (4):

% inhibition of parasites = 100 − (% parasites)(4)

Results of antileishmanial activities were expressed as the EC_50_ calculated by Probit method. The compound were classified according to EC_50_ values using a scale defined by us, based on the hit criteria proposed by Katsuno et al.[25]. Thus, compounds with EC_50_ < 10 μM were classified as highly active while compounds with EC_50_ > 10 μM and <50 μM were defined as moderately active. In turn, compounds were defined with low activity when EC_50_ was >50 μM.

## 5. Conclusions

From the comparison of the biological data of the thiochroman-4-ones and the ones of their hydrazone derivatives, we can assess that the presence of a hydrazone moiety is crucial for the antileishmanial activity. A strong improvement in the biological activity was observed by the introduction of the phenyl group (thioflavanone) with respect to thiochroman-4-one. Two of the compounds stood out as promising antiparasitic agents possessing an activity below 10 µM. These results demonstrated the promising potential of this hydrazone-thiochroman scaffold for the development of new antileishmanial drugs. The mechanism of action of these compounds needs to be addressed in order to optimize the activity. Further studies on animal models of leishmaniasis are needed to confirm the in vitro results.

## Figures and Tables

**Table 1:** In vitro antileishmanial and cytotoxic activities.

Compound	R_1_	R_2_	R_3_	X	EC_50_ (µM) ^a^	LC_50_ (µM) ^a^	IS ^b^
**1**	H	H	-	-	343.8 ± 75.6	>1000	<2.9
**2**	F	H	-	-	>109.9	>706.0 ± 34.6	<6.5
**3**	H	CH_3_	-	-	444.6 ± 7.3	604.2 ± 86.4	1.4
**4**	F	CH_3_	-	-	422.0 ± 9.2	578.9 ± 59.1	1.4
**5**	H	C_6_H_5_	-	-	44.1 ± 0.9	>41.61	>0.9
**6**	H	H	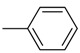	O	63.7 ± 9.2	248.3 ± 49.6	3.9
**7**	H	H	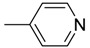	O	56.8 ± 4.54	705.8	>12.4
**8**	H	H	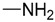	O	91.5 ± 33.4	637.2 ± 37.9	7.0
**9**	H	H	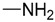	S	55.7 ± 22.1	>842.6	>15.1
**10**	F	H	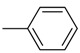	O	37.3 ± 3.3	>665.9	>17.6
**11**	F	H	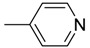	O	39.9 ± 5.3	>663.7	>16.6
**12**	F	H	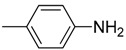	O	95.5 ± 6.9	>634.2	>6.6
**13**	H	CH_3_	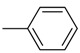	O	38.1 ± 16.2	150.1 ± 24.3	3.9
**14**	H	CH_3_	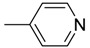	O	56.6 ± 0.7	>672.5	>11.9
**15**	H	CH_3_	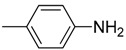	O	91.8 ± 13.5	102.1 ± 15.1	1.1
**16**	F	CH_3_	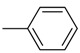	O	43.9 ± 4.5	>636.2	14.5
**17**	F	CH_3_	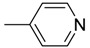	O	98.9 ± 19.3	203.3 ± 25.4	2.1
**18**	F	CH_3_	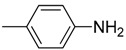	O	160.7 ± 2.4	31.0 ± 7.0	0.2
**19**	H	C_6_H_5_	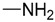	O	5.4 ± 1.0	100.2 ± 19.8	18.6
**20**	H	C_6_H_5_	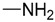	S	5.1 ± 1.3	50.1 ± 4.1	9.8
**21**	H	C_6_H_5_	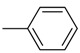	O	28.5 ± 2.8	528.6 ± 7.0	19.6
**22**	H	C_6_H_5_	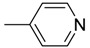	O	16.4 ± 3.6	>556.4	>33.9
thiosemicarbazide					>266.7	>1000	-
Amphotericin B	-	-	-	-	0.32 ± 1.04	39.6 ± 8.7	132.0

^a^ Results reported as the mean value ± standard deviation of the half-maximum concentration in μM. ^b^ Index of Selectivity (IS) = LC_50_/EC_50_. Data in bold indicate high activity.
